# Hyperchloremia and postoperative acute kidney injury: a retrospective analysis of data from the surgical intensive care unit

**DOI:** 10.1186/s13054-018-2216-5

**Published:** 2018-10-30

**Authors:** Tak Kyu Oh, In-Ae Song, Se Joong Kim, Sung Yoon Lim, Sang-Hwan Do, Jung-Won Hwang, Jinhee Kim, Young-Tae Jeon

**Affiliations:** 1 Department of Anesthesiology and Pain Medicine, Seoul National University Bundang Hospital, 166, Gumi-ro, Bundang-gu, Seongnam, 463-707 Republic of Korea; 2 Division of Nephrology, Department of Internal Medicine, Seoul National University Bundang Hospital, Seongnam, Republic of Korea

**Keywords:** Acute kidney injury, Critical care, Hyperchloremia, Intensive care unit

## Abstract

**Background:**

Whether perioperative hyperchloremia can induce postoperative acute kidney injury (AKI) is controversial. We investigated the association between perioperative hyperchloremia and postoperative AKI in patients admitted to the intensive care unit (ICU) after surgery.

**Methods:**

We performed a retrospective observational study of patients admitted to the surgical ICU at a single tertiary care hospital between January 2011 and June 2016. Our primary objective was to determine whether hyperchloremia or an increase in serum chloride levels was associated with postoperative AKI. Perioperative hyperchloremia was defined as serum chloride levels ≥ 110 mmol·L^− 1^ during postoperative days (PODs) 0–3. The increase in serum chloride levels was defined as the difference between preoperative and maximum postoperative serum chloride levels during the first 3 days after surgery.

**Results:**

Of the 7991 patients included in the final analysis, 1876 (23.5%) developed hyperchloremia during PODs 0–3, and 1187 (14.9%) developed postoperative AKI. Exposure to hyperchloremia during the first 3 days after surgery was not associated with postoperative AKI (odds ratio, 1.09; 95% confidence interval, 0.80–1.49; *P* = 0.571). However, among patients with preoperative chronic kidney disease stage ≥ 3 (estimated glomerular filtration rate < 60 mL·min^− 1^·1.73·m^− 2^), the incidence of postoperative AKI was higher in patients with an increase > 6 mmol·L^− 1^ in serum chloride levels than in patients with an increase ≤ 1 mmol·L^− 1^ (odds ratio, 1.42; 95% confidence interval, 1.09–1.84; *P* = 0.009). In addition, the incidence of postoperative AKI stage ≥ 2 was not associated with exposure to hyperchloremia or with the increase in serum chloride levels during PODs 0–3, regardless of preoperative kidney function.

**Conclusions:**

Exposure to perioperative hyperchloremia is not associated with postoperative AKI in surgical ICU patients. However, in patients with moderate-to-severe chronic kidney disease (stage ≥ 3), a substantial perioperative increase in serum chloride levels may reflect a higher risk of AKI.

**Electronic supplementary material:**

The online version of this article (10.1186/s13054-018-2216-5) contains supplementary material, which is available to authorized users.

## Background

Acute kidney injury (AKI) is characterized by elevated serum creatinine and/or decreased urine output due to a sudden loss of renal function [1]. Critically ill patients are particularly at risk, accounting for 57% of AKI cases [2–4]. AKI that occurs during the perioperative period is associated with progression to chronic kidney disease (CKD), heart failure, stroke, and postoperative mortality [5].

Hyperchloremic acidosis is a potential risk factor for AKI after abdominal surgery [6], septic shock [7], and subarachnoid hemorrhage [8]. Perioperative hyperchloremia is also associated with postoperative mortality after non-cardiac surgery [9]. However, the association between hyperchloremia and postoperative AKI in patients who have undergone specific types of surgery remains unclear, as does the association between hyperchloremia and postoperative AKI in patients admitted to the surgical intensive care unit (ICU) postoperatively.

We investigated the association between perioperative hyperchloremia and AKI in patients admitted to the ICU postoperatively. We included all surgical patients, regardless of surgery type and of the presence of CKD preoperatively. We hypothesized that the incidence of postoperative AKI would be higher among patients with hyperchloremia.

The primary objective of this study was to determine whether perioperative hyperchloremia was associated with postoperative AKI. The secondary objective was to examine whether a perioperative increase in serum chloride (Cl^−^) was associated with postoperative AKI. We also examined whether the presence of CKD affected the relationship between perioperative hyperchloremia and postoperative AKI.

## Methods

### Patients and study design

This retrospective observational study was approved by the Institutional Review Board (IRB) of the Seoul National University Bundang Hospital (SNUBH) (approval number B-1806/474-105). The requirement for written informed consent was waived by the IRB. The study included all adult patients (≥ 19 years old) admitted to SNUBH between 1 January 2011 and 30 June 2016, who were admitted to the surgical ICU after undergoing surgical procedures. If a patient was admitted to the surgical ICU more than once during the study period, only the last postoperative ICU admission was considered. The exclusion criteria were as follows: (1) lack of accurate records of main perioperative laboratory investigations; (2) death within 72 h of postoperative ICU admission; (3) preoperative AKI; or (4) receiving chronic renal replacement therapy before surgery.

As of August 2017, SNUBH is a 1360-bed tertiary care hospital with five ICUs (medical, surgical, neurologic, and emergency I and II). During the study period, surgical ICU admission was indicated according to the complexity of the surgery and the severity of the patient’s condition. The decision on ICU admission was made by the intensivist, who was the main researcher (IAS) in the present study.

### Diagnosis of postoperative AKI

For the diagnosis of postoperative AKI, we employed the criteria and grading system laid out in the Kidney Disease: Improving Global Outcomes (KDIGO) guidelines [10] because the KDIGO classification is recognized as the most adequate tool for this purpose [11, 12]. Specifically, AKI diagnosis was based only on creatinine levels (Additional file 1). Creatinine levels were obtained by venous measurement; the most recent measurement obtained within 1 month before surgery was considered as the baseline value. Postoperative AKI was defined as AKI diagnosed within 3 days postoperatively. During the study period, postoperative AKI was diagnosed by certified nephrologists; for the purpose of this study, two certified intensivists (TKO and IAS) reviewed all AKI diagnoses to confirm postoperative AKI. If there were disagreements between the two certified intensivists, the final decision was made through consultation with the nephrologists.

### Data collection and outcomes

Demographic, clinical characteristics, and laboratory test results were obtained by retrospective review of medical charts. At SNUBH, samples for measuring serum creatinine and electrolyte levels are collected during routine preoperative laboratory testing. For the purpose of this study, the baseline creatinine and electrolyte (chloride) levels were defined as the levels measured most recently in blood sampled from the vein within 1 month before surgery. For the diagnosis of postoperative AKI, creatinine levels were measured in samples collected on postoperative days (PODs) 0–3. To identify patients with CKD, we obtained the preoperative estimated glomerular filtration rate (eGFR, mL·min^− 1^·1.73·m^− 2^) using the Modification of Diet in Renal Disease formula [13]. We recorded information on perioperative fluid management, including the type and dosage of fluid infused: NaCl 0.9% (mL·kg^− 1^), NaCl 0.45% (mL·kg^− 1^), balanced electrolyte solution (Ringer’s lactate or Plasmalyte; mL·kg^− 1^), colloid (hydroxyethyl starch; mL·kg^− 1^), red blood cell infusion (packs), and free water-containing dextrose (mL·kg^− 1^). The use of relevant drugs during PODs 0–3 was also recorded: inotropes or vasopressors (norepinephrine, vasopressin, dopamine, dobutamine, epinephrine), diuretics (mannitol, furosemide), radiocontrast agents, nephrotoxic antibiotics (aminoglycoside, cephalosporin, vancomycin, and sulfonamide), and nonsteroidal anti-inflammatory drugs. Data on invasive and non-invasive measurement of intraoperative blood pressure were collected, and intraoperative hypotension was defined as intraoperative mean blood pressure < 60 mmHg for > 1 min. Intraoperative fluid balance (percent) was calculated using the following formula: [total input fluid (L) – total output fluid (L)] × 100 × [weight on admission (kg)]^− 1^.

As described previously [9], hyperchloremia was defined as postoperative Cl^−^ > 110 mmol·L^− 1^ measured at least once during PODs 0–3. Exposure to hyperchloremia on the day of surgery (POD 0) was recorded as an additional independent variable. The largest (maximum) value of Cl^−^ measured during PODs 0–3 was also retained. The increase in Cl^−^ was computed as follows the maximum Cl^−^ or sodium levels during PODs 0–3 minus the preoperative Cl^−^. Metabolic acidosis was diagnosed in patients with simultaneous presence of pH < 7.35 and HCO_3_^−^ < 24 mEq·L^− 1^ on arterial blood gas analysis during PODs 0–3.

SNUBH medical record technicians blinded to the study goals collected all electronic medical record data. During data collection (i.e., until the statistical analysis was performed), the main researchers were blinded to data on Cl^−^, which represent the most important data from the perspective of this study.

### Statistical analysis

Patients were stratified into four groups according to baseline eGFR (≥ 90, 60–89, 30–60, and < 30 mL·min^− 1^·1.73·m^− 2^), incidence of hyperchloremia during PODs 0–3, increase in serum Cl^−^ (quartiles Q1–Q4) during PODs 0–3, and incidence of AKI (yes/no) during PODs 0–3, as explained in detail subsequently. Continuous variables were analyzed using the *t* test, while categorical variables were analyzed using the chi-square test.

We tested the potential association between postoperative AKI and exposure to perioperative hyperchloremia and increase in Cl^−^. Restricted cubic splines (RCS) were used to analyze the relationship between the probability of postoperative AKI and either maximum Cl^−^ or increase in Cl^−^ perioperatively. Since the relationship between the probability of occurrence of postoperative AKI and the increase in Cl^−^ was not linear and the third quartile (Q3) of the distribution of values for the increase in Cl^−^ was close to an inflection point where the slope of the RCS graph changed, the distribution of values of increase in Cl^−^ was divided into quartiles, and the increase in Cl^−^ was analyzed as a categorical variable.

Next, we performed univariable logistic regression analysis for occurrence of postoperative AKI during PODs 0–3. Only covariates with *P* < 0.2 on univariable analysis were included in the multivariable model, with two types of main independent variables (perioperative hyperchloremia as binary exposure; perioperative increase in Cl^−^ as continuous exposure). We generated separate multivariable models for exposure to hyperchloremia and for increase in Cl^−^ during PODs 0–3 to avoid multi-collinearity between the two models.

Because CKD is a well-known risk factor for AKI [14], we investigated the interaction between preoperative eGFR (normal kidney function, > 90 mL·min^− 1^·1.73·m^− 2^; CKD stage 2, 60–89 mL·min^− 1^·1.73·m^− 2^; CKD stage 3, 30–60 mL·min^− 1^·1.73·m^− 2^; CKD stage 4 or 5, < 30 mL·min^− 1^·1.73·m^− 2^) and the two types of main independent variables (hyperchloremia as binary exposure; increase in Cl^−^ as continuous exposure). When interaction between preoperative eGFR and the main independent variables was noted, we performed three additional subgroup analyses. In these subgroup analyses, we performed Bonferroni correction to control type 1 error, and a Bonferroni-corrected *P* value <0.013 was considered to indicate statistical significance. The same method of analysis as described above was applied for postoperative AKI stage ≥ 2 as a dependent variable. For all multivariable models, the goodness of fit was confirmed using the Hosmer-Lemeshow test.

Additionally, Pearson correlation analysis was performed to evaluate the simple relationship between Cl^−^ and infusion of various fluids during PODs 0–3. All analyses were performed using IBM SPSS (version 24.0; IBM Corp., Armonk, NY, USA) and R (version 3.3.3; various R packages; http://www.r-project.org), with statistical significance set at *P* < 0.05 for group analyses and at a Bonferroni-corrected *P* value <0.013 for subgroup analyses.

## Results

### Patients

**Fig. 1:**
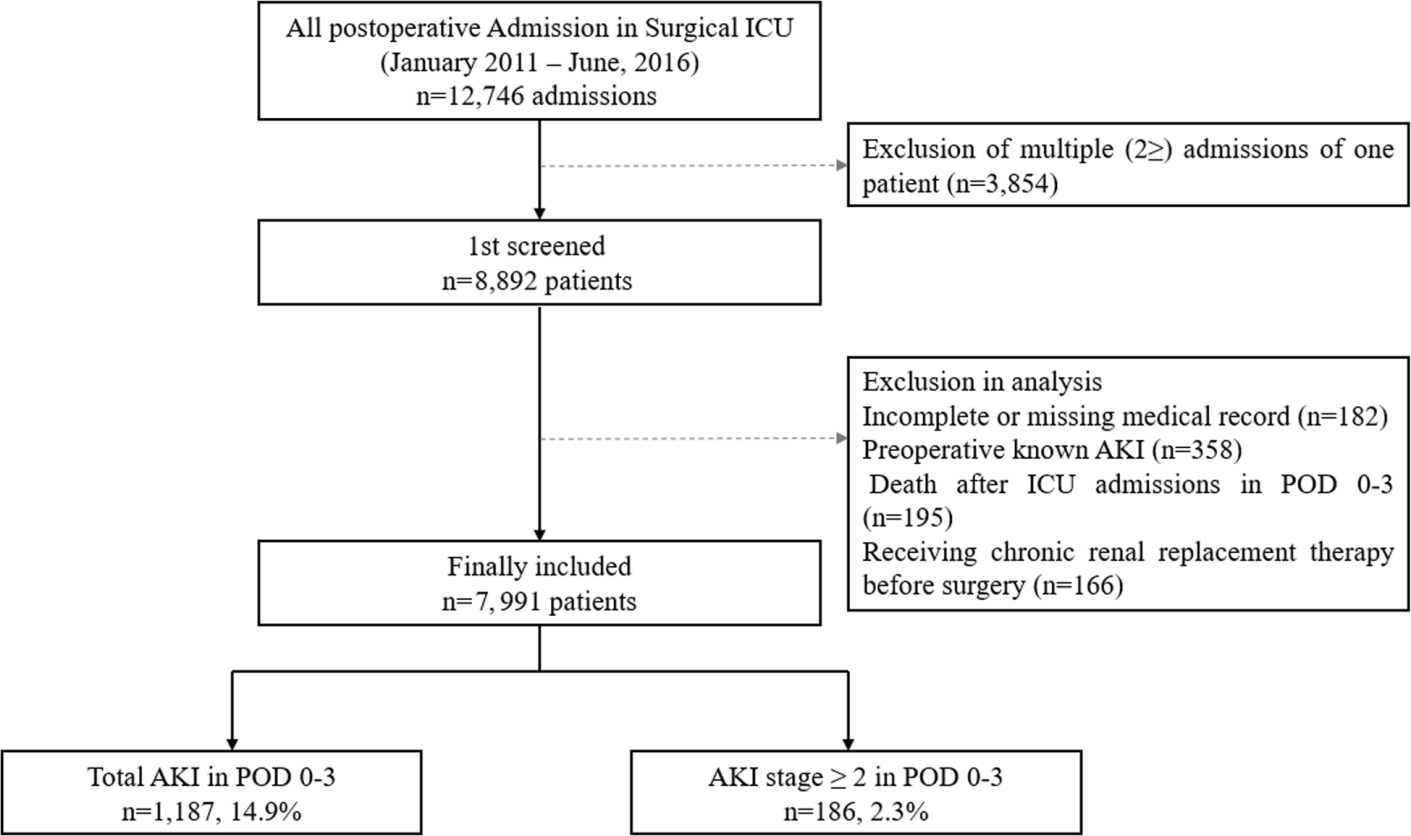
Flowchart of all postoperative ICU admissions. ICU, intensive care unit; POD, postoperative day

Between 1 January 2011 and 30 June 2016, 12,746 patients were admitted to the ICU following surgery. Of these, 3854 postoperative ICU admissions were excluded because they represented multiple admissions; only the last ICU admission was considered for each patient. Additionally, 182 patients were excluded due to incomplete medical records, 358 patients due to preoperative AKI, 195 patients due to death within 72 h of ICU admission, and 166 due to receiving chronic renal replacement therapy before surgery. In total, 7991 patients were included in the final analysis. Of these, 1187 patients (14.9%) were diagnosed with postoperative AKI, of whom 186 (2.3%) exhibited AKI of stage ≥ 2 (Fig. 1). Table 1 summarizes the differences between patients with hyperchloremia and those with non-hyperchloremia during PODs 0–3. The two groups did not differ significantly in the incidence of postoperative AKI (289/1876, 15.4% vs 898/6115, 14.7%; *P* = 0.443) or AKI of stage ≥ 2 (51/1876, 2.7% vs 135/6276, 2.2%; *P* = 0.199).

**Table 1:** Characteristics of patients with and without perioperative hyperchloremia (Cl^−^ ≥ 110 mmol·L^− 1^)

Variables	Perioperative hyperchloremia (*n* = 1876, 23.5%)	No perioperative hyperchloremia (*n* = 6115, 76.5%)	*P* value
Preoperative characteristics			
Male sex	1091 (58.2%)	3636 (59.5%)	0.315
Age, years	58.0 (15.7)	62.1 (15.6)	<0.001
Body mass index, kg m^−2^	23.5 (3.6)	23.6 (3.8)	0.240
Surgery time, min	268.8 (150.9)	220.1 (147.6)	<0.001
ASA class			<0.001
1	433 (23.1%)	975 (15.9%)	
2	892 (47.5%)	3191 (52.2%)	
≥ 3	551 (29.4%)	1949 (31.9%)	
Cancer	405 (21.6%)	1840 (30.1%)	<0.001
eGFR*^a^, mL·min^− 1^·1.73·m^−2^			0.313
≥ 90	841 (44.8%)	2596 (42.5%)	
60–89	528 (28.1%)	1825 (29.8%)	
30–60	310 (16.5%)	1029 (16.8%)	
< 30	197 (10.5%)	665 (10.9%)	
Intraoperative characteristics			
Type of operation			<0.001
Non-cardiovascular surgery	1567 (83.5%)	4786 (78.3%)	
Cardiovascular surgery	309 (16.5%)	1329 (21.7%)	
Emergency surgery	329 (17.5%)	781 (12.8%)	<0.001
Intraoperative hypotension^b^	455 (24.3%)	1659 (27.1%)	0.013
Type of anesthesia			<0.001
General anesthesia	1823 (97.5%)	5474 (89.9%)	
Regional anesthesia	23 (1.2%)	278 (4.6%)	
Monitored anesthesia care	24 (1.3%)	335 (5.5%)	
Patient management (PODs 0–3)			
NaCl 0.9% infused, mL kg^−1^	12.3 (13.2)	8.4 (9.5)	<0.001
NaCl 0.45% infused, mL kg^− 1^	6.5 (20.3)	2.9 (12.9)	<0.001
Balanced electrolyte solution infused, mL kg^−1^	41.9 (37.1)	49.7 (40.7)	<0.001
Free water containing dextrose, mL kg^−1^	47.2 (62.5)	59.3 (56.9)	<0.001
Hydroxyethyl starch infused, mL kg^−1^	22.1 (21.5)	13.4 (16.5)	<0.001
Intraoperative fluid balance, %^c^	1.8 (3.6)	1.7 (2.8)	0.321
RRT (without RRT history)	51 (2.7%)	135 (2.2%)	0.199
Use of inotropes/vasopressors^d^	1635 (87.2%)	4286 (70.1%)	<0.001
Use of diuretics^e^	1300 (69.3%)	3423 (56.0%)	<0.001
Use of radiocontrast	991 (52.8%)	1738 (28.4%)	<0.001
Use of nephrotoxic antibiotics^f^	377 (20.1%)	1147 (18.8%)	0.197
Use of NSAIDs	754 (40.2%)	2194 (35.9%)	0.001
Postoperative laboratory (PODs 0–3) and clinical outcomes			
Maximum Cl^−^, mmol/L*	112.3 (2.9)	104.8 (3.3)	<0.001
Increase in Cl^−^, mmol/L**	5.6 (4.4)	3.5 (3.2)	<0.001
Postoperative metabolic acidosis (PODs 0–3)	262 (14.0%)	615 (10.1%)	<0.001
AKI occurrence	289 (15.4%)	898 (14.7%)	0.443
AKI stage ≥ 2 occurrence	51 (2.7%)	135 (2.2%)	0.199

Values are expressed as the mean (standard deviation) or number (percentage)

*ASA* American Society of Anesthesiologists classification, *eGFR* estimated glomerular filtration rate, *RRT* renal replacement therapy, *POD* postoperative day, *NSAID* nonsteroidal anti-inflammatory drug; AKI, acute kidney injury

*Laboratory values within 3 days after operation

**The increase in Cl^−^ or serum sodium levels was calculated as the difference between the preoperative value and the maximum value noted during PODs 0–3

^a^eGFR (mL·min^− 1^·1.73·m^− 2^) = 186 × (Creatinine)^-1.154^ × (Age)^-0.203^ (× 0.742 if female)

^b^Intraoperative hypotension was defined as mean blood pressure < 60 mmHg for > 1 min

^c^Intraoperative fluid balance (%) = {[Total input fluid (L) – Total output fluid (L)] × 100} × weight on admission^− 1^ (kg)

^d^Inotropes/vasopressors include norepinephrine, epinephrine, vasopressin, dobutamine, and dopamine

^e^Diuretics include mannitol and furosemide

^f^Nephrotoxic antibiotics include aminoglycoside, cephalosporin, vancomycin, and sulfonamide

### Exposure to hyperchloremia (Cl^−^ ≥ 110 mmol L^− 1^) and postoperative AKI

The RCS describing the relationship between postoperative AKI and maximum Cl^−^ are provided in Additional file 2 (panel A), while the results of the univariable logistic regression analyses are presented in Additional files 3 and 4. The covariates with *P* < 0.2 on the univariable analysis were entered into the multivariable logistic regression model, and the results of multivariable analysis based on model 1 are provided in Table 2. In the overall sample (7991 patients), exposure to hyperchloremia was not associated with postoperative AKI (odds ratio (OR), 1.09; 95% confidence interval (CI), 0.80–1.49, *P* = 0.571; model 1) or with AKI of stage ≥ 2 (OR, 0.77; 95% CI 0.40–1.49; *P* = 0.437; model 2).

**Table 2:** Multivariable logistic regression analyses o postoperative AKI according to hyperchloremia exposure during PODs 0–3

Group	Variable	Multivariable model 1	
		Overall AKI: OR (95% CI)	*P* value
Entire sample (*n* = 7991)	Hyperchloremia^a^ on POD 0	1.22 (0.94, 1.58)	0.144
	Hyperchloremia^a^ on PODs 0–3	1.09 (0.80, 1.49)	0.571
	Interaction of hyperCl on POD 0–3 with eGFR		
	≥ 90 mL·min^−1^·1.73·m^− 2^	1	(0.425)
	60–89 mL·min^−1^·1.73·m^− 2^	0.91 (0.62, 1.34)	0.629
	30–60 mL·min^−1^·1.73·m^− 2^	0.96 (0.65, 1.43)	0.840
	< 30 mL·min^−1^·1.73·m^−2^	0.70 (0.45, 1.08)	0.109
		Multivariable model 2	
		≥ Stage 2 AKI: OR (95% CI)	*P* value
	Hyperchloremia^a^ on POD 0	1.61 (0.89, 2.93)	0.367
	Hyperchloremia^a^ on PODs 0–3	0.77 (0.40, 1.49)	0.437
	Interaction of hyperchloremia with eGFR		
	≥ 90 mL·min^−1^·1.73·m^−2^	1	(0.468)
	60–89 mL·min^− 1^·1.73·m^− 2^	1.40 (0.67, 2.94)	0.367
	30–60 mL·min^− 1^·1.73·m^− 2^	0.64 (0.24, 1.69)	0.363
	< 30 mL·min^− 1^·1.73·m^− 2^	0.91 (0.17, 4.94)	0.915

Covariates with *P* < 0.2 on univariable analysis (see also Additional file 3) were included in the final multivariable model to adjust for potential confounders. Covariates for multivariable model 1 included preoperative American Society of Anesthesiologists (ASA) score, preoperative estimated glomerular filtration rate (eGFR), NaCl 0.9% and balanced crystalloid infused. Covariates for multivariable model 2 included preoperative ASA score, preoperative eGFR, cardiovascular surgery, NaCl 0.45% and hydroxyethyl starch infused. The Hosmer-Lemeshow goodness-of-fit test was performed to investigate the fitness of the multivariable logistic regression model, and revealed no indication of lack of fit (Model 1: Hosmer-Lemeshow statistic 7.09, *P* = 0.527; Model 2: Hosmer-Lemeshow statistic 6.287, *P* = 0.615)

*AKI* acute kidney injury, *CI* confidence interval, *POD* postoperative day

^a^Hyperchloremia was defined as serum chloride levels ≥ 110 mmol·L^− 1^

^b^Preoperative eGFR (mL·min^− 1^·1.73·m^− 2^) was calculated using the Modification of Diet in Renal Disease formula: 186 × (preoperative serum Creatinine)^-1.154^ × (Age)^-0.203^ (× 0.742 if female)

### Increase in Cl^−^ and postoperative AKI

The RCS describing the relationship between postoperative AKI and increase in Cl^−^ are provided in Additional file 1 (panel B). Based on the shape of the RCS curve, the distribution of values for the increase in Cl^−^ was divided into quartiles: Q1, increase in Cl^−^ ≤ 1 mmol·L^− 1^, 2535 patients (31.7%); Q2, increase in Cl^−^ of 1–3 mmol·L^− 1^, 1593 patients (20.0%); Q3, increase in Cl^−^ of 3–6 mmol·L^− 1^, 2075 patients (26.0%); and Q4, increase in Cl^−^ > 6 mmol·L^− 1^, 1788 patients (22.4%).

The results of the multivariable regression analysis of increase in Cl^−^ are provided in Table 3. The overall *P* value for the 4-level factor (Q1–Q4) was 0.756. However, there was interaction between perioperative Cl^−^ increase and preoperative kidney function (Q1 * eGFR ≥ 90 mL·min^− 1^·1.73·m^− 2^ and Q4 * eGFR < 30 mL·min^− 1^·1.73·m^− 2^), and thus subgroup analyses were performed for each predefined eGFR threshold (≥ 90, < 90, < 60, and < 30 mL·min^− 1^·1.73·m^− 2^). On subgroup analysis, a Cl^−^ increase > 6 mmol·L^− 1^ was associated with postoperative AKI in patients with CKD stage ≥ 3 (eGFR < 60 mL·min^− 1^·1.73·m^− 2^) (OR, 1.42; 95% CI, 1.09–1.84; *P* = 0.009 vs a Cl^−^ increase ≤ 1 mmol·L^− 1^). In addition, there was no significant association between the increase in Cl^−^ and occurrence of postoperative AKI of stage ≥ 2, and there was no significant interaction for AKI stage ≥ 2 between perioperative Cl^−^ increase and preoperative kidney function (Table 4).

**Table 3:** Multivariable logistic regression analyses for postoperative AKI according to increase in serum chloride levels (Cl^−^)

Group	Variable	Multivariable model 3	
		Odds ratio (95% CI)	*P* value^**^
Entire sample (*n* = 7991)	Increase in Cl^−^, mmol L^− 1^*		
	Q1: ≤ 1 mmol L^− 1^	1	(0.756)
	Q2: 1–3 mmol L^− 1^	0.84 (0.60, 1.18)	0.316
	Q3: 3–6 mmol L^− 1^	0.89 (0.66, 1.21)	0.456
	Q4: > 6 mmol L^− 1^	0.90 (0.66, 1.24)	0.528
	Interaction of increase in Cl^−^ with eGFR^a^		
	Increase in Cl^−^: Q1 * eGFR: ≥ 90	1	(0.297)
	Increase in Cl^−^: Q2 * eGFR: 60–89	1.04 (0.64, 1.70)	0.867
	Increase in Cl^−^: Q2 * eGFR: 30–60	0.93 (0.60, 1.44)	0.738
	Increase in Cl^−^: Q2 * eGFR: < 30	1.02 (0.60, 1.75)	0.934
	Increase in Cl^−^: Q3 * eGFR: 60–89	0.93 (0.60, 1.44)	0.738
	Increase in Cl^−^: Q3 * eGFR: 30–60	0.99 (0.63, 1.57)	0.973
	Increase in Cl^−^: Q3 * eGFR: < 30	1.10 (0.67, 1.80)	0.702
	Increase in Cl^−^: Q4 * eGFR: 60–89	0.92 (0.58, 1.46)	0.728
	Increase in Cl^−^: Q4 * eGFR: 30–60	1.51 (0.95, 2.40)	0.084
	Increase in Cl^−^: Q4 * eGFR: < 30	1.67 (1.01, 2.77)	0.045
	Four subgroup analysis	Odds ratio (95% CI)	*P* value^***^
eGFR ≥ 90 (*n* = 3437)	Increase in Cl^−^, mmol L^− 1^		
	Q1: ≤ 1 mmol L^− 1^	1	(0.739)
	Q2: 1–3 mmol L^− 1^	0.84 (0.60, 1.19)	0.323
	Q3: 3–6 mmol L^− 1^	0.90 (0.66, 1.22)	0.491
	Q4: > 6 mmol L^− 1^	0.90 (0.66, 1.24)	0.529
eGFR < 90 (*n* = 4554)	Increase in Cl^−^, mmol L^− 1^		
	Q1: ≤ 1 mmol L^− 1^	1	(0.023)
	Q2: 1–3 mmol L^− 1^	0.84 (0.68, 1.05)	0.123
	Q3: 3–6 mmol L^− 1^	0.88 (0.72, 1.07)	0.195
	Q4: > 6 mmol L^− 1^	1.16 (0.94, 1.41)	0.164
eGFR < 60 (*n* = 2201)	Increase in Cl^−^, mmol L^− 1^		
	Q1: ≤ 1 mmol L^− 1^	1	
	Q2: 1–3 mmol L^− 1^	0.82 (0.61, 1.09)	0.164
	Q3: 3–6 mmol L^− 1^	0.90 (0.70, 1.17)	0.430
	Q4: > 6 mmol L^− 1^	1.42 (1.09, 1.84)	0.009
eGFR < 30 (*n* = 862)	Increase in Cl^−^, mmol/L		
	Q1: ≤1 mmol L^− 1^	1	(0.068)
	Q2: 1–3 mmol L^− 1^	0.85 (0.56, 1.28)	0.428
	Q3: 3–6 mmol L^− 1^	0.94 (0.64, 1.40)	0.775
	Q4: > 6 mmol L^− 1^	1.48 (1.00, 2.21)	0.053

Covariates with *P* < 0.2 on univariable analysis (see also Additional file 3) were included in the final multivariable model to adjust for potential confounders. Covariates for multivariable model 3 included preoperative American Society of Anesthesiologists (ASA) score, preoperative estimated glomerular filtration rate (eGFR), NaCl 0.9% and balanced crystalloid infused. The Hosmer-Lemeshow goodness-of-fit test was performed to investigate the fitness of the multivariable logistic regression models, and revealed no indication of lack of fit. Specifically, the Hosmer-Lemeshow statistic and *P* value, respectively, were 4.38 and 0.821 for the entire sample; 4.44 and 0.823 for the group of patients with eGFR ≥ 90 mL·min^− 1^·1.73·m^− 2^; 10.98 and 0.203 for the group of patients with eGFR < 90 mL·min^− 1^·1.73·m^− 2^; 11.92 and 0.155 for the group of patients with eGFR < 60 mL·min^− 1^·1.73·m^− 2^; 5.29 and 0.726 for the group of patients with eGFR < 30 mL·min^− 1^·1.73·m^− 2^

*AKI* acute kidney disease, *CI* confidence interval, *Cl*^*−*^ serum chloride levels

^a^Preoperative eGFR (mL·min^− 1^·1.73·m^− 2^) was calculated using the Modification of Diet in Renal Disease formula: 186 × (preoperative serum Creatinine)^-1.154^ × (Age)^-0.203^ (× 0.742 if female)

*The increase in Cl^−^ was calculated as the difference between the preoperative value and the maximum value noted during postoperative days (PODs) 0–3. The following quartiles were obtained: Q1, 2535 patients (31.7%); Q2, 1593 patients (20.0%); Q3, 2075 patients (26.0%); and Q4, 1788 patients (22.4%)

^**^P < 0.05 ^***^P < 0.012 (after Bonferroni correction) were considered to indicate statistical significance

**Table 4:** Multivariable logistic regression analyses for postoperative AKI stage ≥ 2 according to Cl^−^ increase

Group	Variable	Multivariable model 4	
		Odds ratio (95% CI)	*P* value
Entire sample (*n* = 8157)	Increase in Cl^−^, mmol L^− 1^*		
	Q1: ≤1 mmol L^− 1^	1	(0.802)
	Q2: 1–3 mmol L^− 1^	1.22 (0.65, 2.28)	0.542
	Q3: 3–6 mmol L^− 1^	0.86 (0.46, 1.62)	0.642
	Q4: > 6 mmol L^− 1^	1.00 (0.53, 1.89)	0.992
	Interaction of Cl^−^ increase with eGFR^a^		
	Increase in Cl^−^: Q1 * eGFR: ≥ 90	1	(0.960)
	Increase in Cl^−^: Q2 * eGFR: 60–89	0.62 (0.23, 1.67)	0.348
	Increase in Cl^−^: Q2 * eGFR: 30–60	1.01 (0.31, 3.30)	0.990
	Increase in Cl^−^: Q2 * eGFR: < 30	0.00 (0.00)	0.996
	Increase in Cl^−^: Q3 * eGFR: 60–89	1.14 (0.46, 2.79)	0.780
	Increase in Cl^−^: Q3 * eGFR: 30–60	1.44 (0.48, 4.37)	0.518
	Increase in Cl^−^: Q3 * eGFR: < 30	2.52 (0.38, 16.95)	0.342
	Increase in Cl^−^: Q4 * eGFR: 60–89	0.96 (0.38, 2.44)	0.937
	Increase in Cl^−^: Q4 * eGFR: 30–60	1.52 (0.50, 4.62)	0.460
	Increase in Cl^−^: Q4 * eGFR: < 30	2.53 (0.38, 17.01)	0.341

Covariates with *P* < 0.2 on univariable analysis (see also Additional file 4) were included in the final multivariable model to adjust for potential confounders. Covariates for multivariable model 4 included preoperative American Society of Anesthesiologists (ASA) score, preoperative estimated glomerular filtration rate (eGFR), cardiovascular surgery, NaCl 0.45% and hydroxyethyl starch infused

*The increase in Cl^−^ was calculated as the difference between the preoperative value and the maximum value noted during postoperative days (PODs) 0–3. The following quartiles were obtained: Q1, 2535 patients (31.7%); Q2, 1593 patients (20.0%); Q3, 2075 patients (26.0%); and Q4, 1788 patients (22.4%). The Hosmer-Lemeshow goodness-of-fit test was performed to investigate the fitness of the multivariable logistic regression models, and revealed no indication of lack of fit. For the entire sample, the Hosmer-Lemeshow statistic was 5.76 and the *P* value was 0.674

*AKI* acute kidney injury, *CI* confidence interval, *Cl*^*−*^ serum chloride levels

^a^Preoperative eGFR (mL·min^− 1^·1.73·m^− 2^) was calculated using the Modification of Diet in Renal Disease formula: 186 × (preoperative serum Creatinine)^-1.154^ × (Age)^-0.203^ (× 0.742 if female)

## Discussion

In this retrospective review of 7991 patients, we found that exposure to hyperchloremia was not an independent risk factor for postoperative AKI in patients admitted to the ICU after surgery. This result is consistent with the findings of Yessayan et al. [15], who also observed no significant association between hyperchloremia and AKI within 72 h of ICU admission in patients with sepsis or septic shock. However, while Yessayan et al. excluded patients with CKD stage 5 [15], we did not exclude such patients from our analysis. However, we excluded 195 patients who died within 72 h after surgery, whereas Yessayan et al. included such patients. Our reason for excluding patients who died within 72 h was that such patients might have AKI associated with another critical illness not related to perioperative hyperchloremia, such as multi-organ failure or massive bleeding. Yessayan et al. also noted that AKI was not associated with an increase in Cl^−^ [15]. However, in the present study, we found that patients with CKD stage ≥ 3 who exhibit a Cl^−^ increase > 6 mmol·L^− 1^ during PODs 0–3 have higher risk of postoperative AKI. Our results suggest that the association between perioperative hyperchloremia and occurrence of postoperative AKI may depend in part on the presence of perioperative CKD.

Our findings are in contrast to previous observations that had suggested an association between hyperchloremia and AKI in non-cardiac surgery [9], subarachnoid hemorrhage [8], severe sepsis or septic shock [7], and abdominal surgery [6]. A 2018 trial of balanced salt solutions in critically ill patients also found that use of saline increased the composite outcome of death from any cause, new renal replacement therapy, or persistent renal dysfunction [16]. Two of the above-cited trials examined perioperative data. The study by McCluskey et al. found that hyperchloremia was an independent risk factor for renal dysfunction in non-cardiac surgery [9]. The 2013 study by McCluskey et al. [9] differed from our study in several aspects. First, patients with preoperative kidney dysfunction were excluded, whereas our study included all surgical patients, including those with preoperative CKD. Second, McCluskey et al. used the Risk, Injury, Failure, Loss of kidney function, and End-stage kidney disease (RIFLE) criteria [17], whereas we used the KDIGO criteria to diagnose postoperative AKI. The KDIGO criteria have been shown to be more sensitive for diagnosing AKI and to have more predictive value for in-hospital mortality [18]. Last, McCluskey et al. did not evaluate the postoperative increase in Cl^−^ due to lack of data on preoperative Cl^−^. However, since Cl^−^ is routinely measured preoperatively in most tertiary care hospitals in South Korea, we could perform a detailed analysis of the influence of Cl^−^ levels on AKI incidence. Other studies have also identified the increase in Cl^−^ as an important factor for occurrence of AKI [8, 15, 19].

Our results also differ from the findings of a recent study by Toyonaga and Kikura, involving a small cohort of 206 patients admitted to the ICU after elective abdominal surgery [6]. Specifically, Toyonaga and Kikura reported that hyperchloremic acidosis was associated with an increased incidence of AKI; however, they excluded patients with preoperative CKD [6]. On the other hand, we included patients with preoperative CKD and adjusted for CKD in the multivariable logistic analysis. In addition, several studies that reported an association between hyperchloremia and AKI in patients with major trauma [20], subarachnoid hemorrhage [8], severe sepsis or septic shock [7], or intracerebral hemorrhage [21] included patients who received large volumes of NaCl 0.9%. Our study differs from those previous investigations in that we included patients who were admitted to the ICU after undergoing any type of surgery.

Unlike many prior studies of perioperative AKI [7, 8], ours included patients with preoperative CKD. It is currently recognized that preoperative CKD is itself a risk factor for postoperative AKI [22], may predispose to chronic renal insufficiency, and may be involved in the relationship between chloride homeostasis and perioperative AKI. Our study revealed a potential association between a perioperative Cl^−^ increase > 6 mmol·L^− 1^ and the risk of postoperative AKI in patients with CKD stage ≥ 3. These data suggest that perioperative hyperchloremic acidosis may have a greater impact on postoperative renal function in patients already at risk.

Our study has several limitations. First, because of the retrospective design, we could not fully exclude selection bias, especially with respect to the choice of infusion fluid. However, we found no effect of hyperchloremia on postoperative AKI incidence, suggesting that any effect of selection bias was likely modest. Second, our study was conducted in a single center, which limits the generalizability of our findings. Third, postoperative Cl^−^ were measured once per day, but not always at the same time, mainly due to aspects related to the patient’s physical condition. Fourth, there is a possibility that AKI may have preceded the observed change in chloride levels, which would have induced some bias in our analysis.

In particular, because our study was retrospective in nature, we could not control the choice and dosage of infusion fluid during PODs 0–3. With the exception of balanced crystalloid and free water-containing dextrose, most types of infusion fluids correlated positively with the maximum Cl^−^ noted during PODs 0–3 (Additional file 5), suggesting that postoperative AKI incidence was affected not only by Cl^−^ but also by the choice of fluid therapy. The effect of fluid type on AKI incidence is unclear, with two very recent studies reporting an adverse effect of high-chloride solutions in critically ill patients but no effect in non-critically ill patients [16, 23].

## Conclusions

Despite its limitations, this retrospective study of 7991 patients admitted to the ICU after surgery found that exposure to perioperative hyperchloremia is not significantly associated with postoperative AKI. However, in patients with moderate-to-severe chronic kidney disease (stage ≥ 3), a substantial increase in Cl^−^ (> 6 mmol·L^− 1^) was associated with an increased risk of postoperative AKI.

## Additional files

Additional file 1: Staging of postoperative acute kidney injury. Brief overview of KDIGO-based staging criteria for acute kidney injury. (DOCX 29 kb)

Additional file 2: Probability of AKI during the stay in the surgical ICU. AKI probability according to maximum serum chloride levels (A) and according to the increase in serum chloride levels (B) during PODs 0–3. Representation based on restricted cubic splines. AKI, acute kidney injury; ICU, intensive care unit; POD, postoperative day. (ZIP 74 kb)

Additional file 3: Risk factors for AKI in the surgical ICU. Results of the univariable logistic regression analysis for occurrence of AKI after postoperative ICU admission. (DOCX 39 kb)

Additional file 4: Risk factors for AKI stage ≥2 in the surgical ICU. Results of the univariable logistic regression analysis for occurrence of AKI stage ≥2 after postoperative ICU admission. (DOCX 39 kb)

Additional file 5: Association of total fluid use with perioperative serum chloride levels in surgical ICU patients. Results of the Pearson correlation analysis between total fluid use and maximum serum chloride levels or increase in serum chloride levels during PODs 0–3. (DOCX 31 kb)

## Abbreviations

AKI: Acute kidney injury
CKD: Chronic kidney disease
eGFR: Estimated glomerular filtration rate
ICU: Intensive care unit
IRB: Institutional review board
KDIGO: Kidney Disease: Improving Global Outcomes (KDIGO)
POD: Postoperative day
RCS: Restricted cubic spline
SNUBH: Seoul National University Bundang Hospital

## References

[1] Waikar SS, Bonventre JV (2009). Creatinine kinetics and the definition of acute kidney injury. J Am Soc Nephrol.

[2] Bellomo R, Kellum JA, Ronco C (2012). Acute kidney injury. Lancet.

[3] Lewington AJ, Cerda J, Mehta RL (2013). Raising awareness of acute kidney injury: a global perspective of a silent killer. Kidney Int.

[4] Nash K, Hafeez A, Hou S (2002). Hospital-acquired renal insufficiency. Am J Kidney Dis.

[5] Palant CE, Amdur RL, Chawla LS (2017). Long-term consequences of acute kidney injury in the perioperative setting. Curr Opin Anaesthesiol.

[6] Toyonaga Y, Kikura M (2017). Hyperchloremic acidosis is associated with acute kidney injury after abdominal surgery. Nephrology (Carlton).

[7] Suetrong B, Pisitsak C, Boyd JH, Russell JA, Walley KR (2016). Hyperchloremia and moderate increase in serum chloride are associated with acute kidney injury in severe sepsis and septic shock patients. Crit Care.

[8] Sadan O, Singbartl K, Kandiah PA, Martin KS, Samuels OB (2017). Hyperchloremia is associated with acute kidney injury in patients with subarachnoid hemorrhage. Crit Care Med.

[9] McCluskey SA, Karkouti K, Wijeysundera D, Minkovich L, Tait G, Beattie WS (2013). Hyperchloremia after noncardiac surgery is independently associated with increased morbidity and mortality: a propensity-matched cohort study. Anesth Analg.

[10] Kellum JA, Lameire N, Group KAGW (2013). Diagnosis, evaluation, and management of acute kidney injury: a KDIGO summary (Part 1). Crit Care.

[11] Pan HC, Chien YS, Jenq CC, Tsai MH, Fan PC, Chang CH, Chang MY, Tian YC, Fang JT, Yang CW (2016). Acute Kidney Injury Classification for Critically Ill Cirrhotic Patients: a Comparison of the KDIGO, AKIN, and RIFLE Classifications. Sci Rep.

[12] Ülger F., Pehlivanlar Küçük M., Küçük A. O., İlkaya N. K., Murat N., Bilgiç B., Abanoz H. (2017). Evaluation of acute kidney injury (AKI) with RIFLE, AKIN, CK, and KDIGO in critically ill trauma patients. European Journal of Trauma and Emergency Surgery.

[13] Kilbride HS, Stevens PE, Eaglestone G, Knight S, Carter JL, Delaney MP, Farmer CK, Irving J, O'Riordan SE, Dalton RN (2013). Accuracy of the MDRD (Modification of Diet in Renal Disease) study and CKD-EPI (CKD Epidemiology Collaboration) equations for estimation of GFR in the elderly. Am J Kidney Dis.

[14] Chawla LS, Eggers PW, Star RA, Kimmel PL (2014). Acute kidney injury and chronic kidney disease as interconnected syndromes. N Engl J Med.

[15] Yessayan L, Neyra JA, Canepa-Escaro F, Vasquez-Rios G, Heung M, Yee J, Acute Kidney Injury in Critical Illness Study Group (2017). Effect of hyperchloremia on acute kidney injury in critically ill septic patients: a retrospective cohort study. BMC Nephrol.

[16] Semler MW, Self WH, Wanderer JP, Ehrenfeld JM, Wang L, Byrne DW, Stollings JL, Kumar AB, Hughes CG, Hernandez A (2018). Balanced crystalloids versus saline in critically ill adults. N Engl J Med.

[17] Bellomo R, Kellum JA, Ronco C (2007). Defining and classifying acute renal failure: from advocacy to consensus and validation of the RIFLE criteria. Intensive Care Med.

[18] Luo X, Jiang L, Du B, Wen Y, Wang M, Xi X, Beijing Acute Kidney Injury Trial Workgoup (2014). A comparison of different diagnostic criteria of acute kidney injury in critically ill patients. Crit Care.

[19] de Vasconcellos K, Skinner DL (2018). Hyperchloraemia is associated with acute kidney injury and mortality in the critically ill: a retrospective observational study in a multidisciplinary intensive care unit. J Crit Care.

[20] Lee JY, Hong TH, Lee KW, Jung MJ, Lee JG, Lee SH (2016). Hyperchloremia is associated with 30-day mortality in major trauma patients: a retrospective observational study. Scand J Trauma Resusc Emerg Med.

[21] Riha Heidi M., Erdman Michael J., Vandigo Joseph E., Kimmons Lauren A., Goyal Nitin, Davidson K. Erin, Pandhi Abhi, Jones G. Morgan (2017). Impact of Moderate Hyperchloremia on Clinical Outcomes in Intracerebral Hemorrhage Patients Treated With Continuous Infusion Hypertonic Saline. Critical Care Medicine.

[22] Chawla LS, Kimmel PL (2012). Acute kidney injury and chronic kidney disease: an integrated clinical syndrome. Kidney Int.

[23] Self WH, Semler MW, Wanderer JP, Wang L, Byrne DW, Collins SP, Slovis CM, Lindsell CJ, Ehrenfeld JM, Siew ED (2018). Balanced crystalloids versus saline in noncritically ill adults. N Engl J Med.

