# Supplementary material for: Hyperchloremia and postoperative acute kidney injury: a retrospective analysis of data from the surgical intensive care unit
Source: Crit Care. 2018 Oct 30;22:277. doi: 10.1186/s13054-018-2216-5 (PMC6206638; doi:10.1186/s13054-018-2216-5)
Supplement: Supplementary file 1 — Staging of postoperative acute kidney injury. Brief overview of KDIGO-based staging criteria for acute kidney injury. (DOCX 29 kb) [file 13054_2018_2216_MOESM1_ESM.docx]

File name: **Additional file 1**

File format: .docx

Title of data: Staging of postoperative acute kidney injury

Description of data: Brief overview of KDIGO-based staging criteria for acute kidney injury.

| Stage | Serum Creatinine |
| --- | --- |
| 1 | 1.5-1.9 times baseline within one week after surgery or ≥ 0.3 mg dl^-1^ increase within 3 days after surgery |
| 2 | 2.0-2.9 times baseline within one week after surgery |
| 3 | 3.0 times baseline or increase in serum creatinine to ≥ 4.0 mg dl^-1^ within one week after surgery or initiation of RRT |

KDIGO, Kidney Disease: Improving Global Outcomes; RRT, Renal Replacement Therapy
