# Supplementary figures and images for: Hyperchloremia and postoperative acute kidney injury: a retrospective analysis of data from the surgical intensive care unit
Source: Crit Care. 2018 Oct 30;22:277. doi: 10.1186/s13054-018-2216-5 (PMC6206638; doi:10.1186/s13054-018-2216-5)

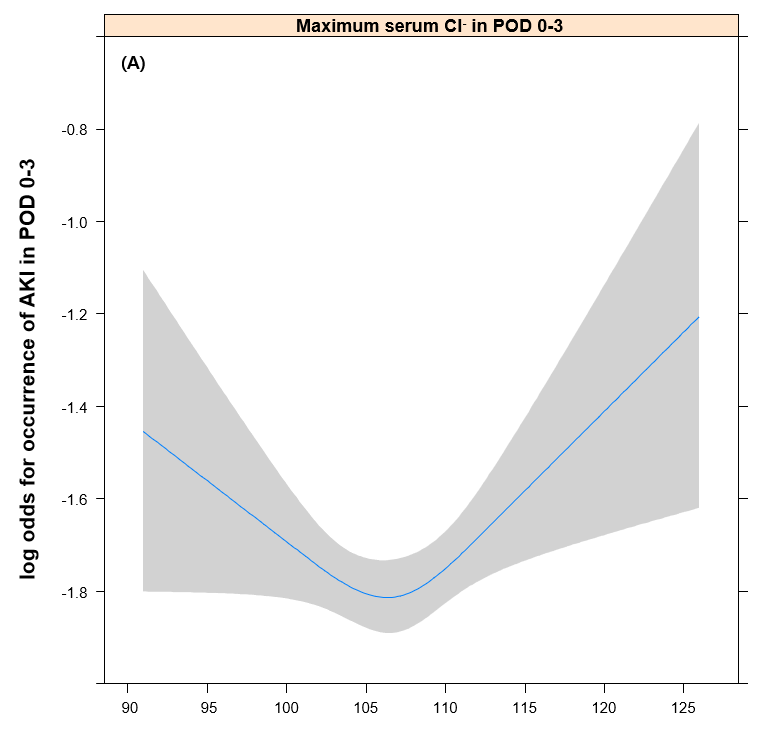

Supplement: Supplementary file 2 — Probability of AKI during the stay in the surgical ICU. AKI probability according to maximum serum chloride levels (A) and according to the increase in serum chloride levels (B) during PODs 0–3. Representation based on restricted cubic splines. AKI, acute kidney injury; ICU, intensive care unit; POD, postoperative day. (ZIP 74 kb) [file 13054_2018_2216_MOESM2_ESM.zip › FigA2A.TIF]

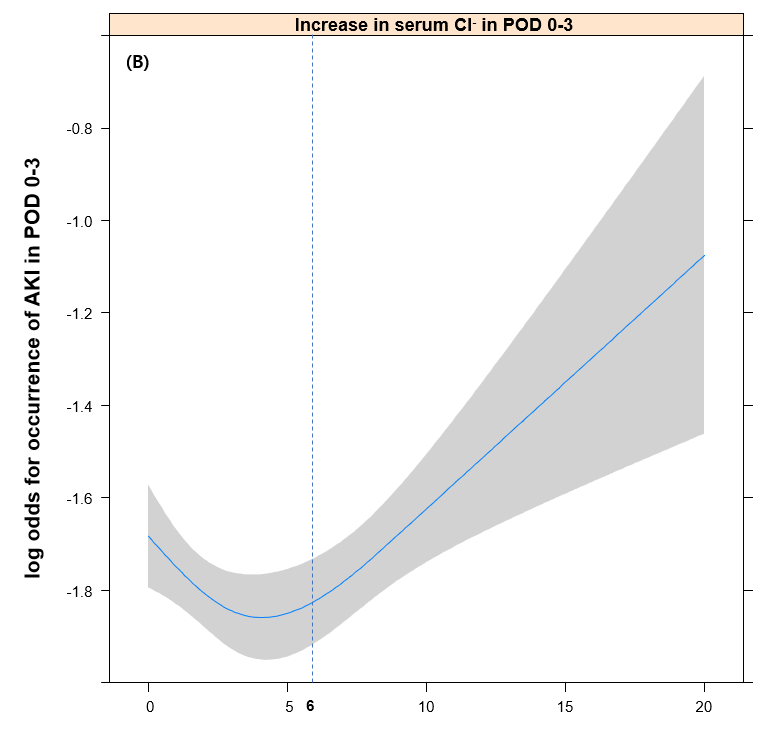

Supplement: Supplementary file 2 — Probability of AKI during the stay in the surgical ICU. AKI probability according to maximum serum chloride levels (A) and according to the increase in serum chloride levels (B) during PODs 0–3. Representation based on restricted cubic splines. AKI, acute kidney injury; ICU, intensive care unit; POD, postoperative day. (ZIP 74 kb) [file 13054_2018_2216_MOESM2_ESM.zip › FigA2B.TIF]
