# Supplementary material for: Hyperchloremia and postoperative acute kidney injury: a retrospective analysis of data from the surgical intensive care unit
Source: Crit Care. 2018 Oct 30;22:277. doi: 10.1186/s13054-018-2216-5 (PMC6206638; doi:10.1186/s13054-018-2216-5)
Supplement: Supplementary file 4 — Risk factors for AKI stage ≥2 in the surgical ICU. Results of the univariable logistic regression analysis for occurrence of AKI stage ≥2 after postoperative ICU admission. (DOCX 39 kb) [file 13054_2018_2216_MOESM4_ESM.docx]

File name: **Additional file 4**

File format: .docx

Title of data: Risk factors for AKI stage ≥ 2 in the surgical ICU

Description of data: Results of the univariable logistic regression analysis for occurrence of AKI stage ≥ 2 after postoperative ICU admission.

| Variables | | | Univariable model | |
| --- | --- | --- | --- | --- |
|  |  |  | Odds ratio (95% CI) | *P*-value |
| Sex: male | | | 0.98 (0.73, 1.31) | 0.877 |
| Age, year | | | 1.00 (0.99, 1.01) | 0.839 |
| Body mass index, kg m^-2^ | | | 1.00 (0.96, 1.04) | 0.831 |
| Preoperative ASA classification | | |  |  |
|  | 1 | | 1 | (0.275) |
|  | 2 | | 0.76 (0.52, 1.10) | 0.150 |
|  | ≥ 3 | | 0.74 (0.49, 1.11) | 0.144 |
| Cancer | | | 1.08 (0.78, 1.48) | 0.651 |
| Preoperative eGFR*^a^, mL·min^-1^·1.73·m^-2^ | | |  |  |
|  | ≥90 | | 1 | (0.032) |
|  | 60–89 | | 1.17 (0.84, 1.64) | 0.360 |
|  | 30-60 | | 1.17 (0.79, 1.75 | 0.430 |
|  | <30 | | 0.40 (0.19, 0.83) | 0.014 |
| Surgery time, hour | | | 1.03 (0.97, 1.09) | 0.359 |
| Type of surgery | | |  |  |
|  | Non-cardiovascular surgery | | 1 |  |
|  | Cardiovascular surgery | | 0.62 (0.41, 0.95) | 0.027 |
| Emergency surgery | | | 1.10 (0.73, 1.65) | 0.643 |
| Intraoperative hypotension^b^ | | | 1.01 (0.95, 1.07) | 0.814 |
| Type of anesthesia | | |  |  |
|  | General anesthesia | | 1 |  |
|  | Regional anesthesia | | 0.99 (0.46, 2.13) | 0.984 |
|  | Monitored anesthesia care | | 0.71 (0.31, 1.61) | 0.410 |
| Patient management (PODs 0–3) | | |  |  |
|  | NaCl 0.9% infused, mL kg | | 1.00 (0.98, 1.01) | 0.842 |
|  | NaCl 0.45% infused, mL kg^-1^ | | 1.01 (1.00, 1.01) | 0.106 |
|  | Balanced electrolyte solution infused, mL kg^-1^ | | 1.00 (1.00, 1.00) | 0.772 |
|  | Free water containing dextrose, mL kg^-1^ | | 1.00 (1.00, 1.00) | 0.438 |
|  | Hydroxyethyl starch infused, mL kg^-1^ | | 1.01 (1.00, 1.02) | 0.010 |
|  | Intraoperative fluid balance, %^c^ | | 1.01 (0.95, 1.07) | 0.814 |
|  | Use of inotropes/vasopressors^d^ | | 1.07 (0.76, 1.49) | 0.712 |
|  | Use of diuretics^e^ | | 0.90 (0.67, 1.20) | 0.457 |
|  | Use of radiocontrast | | 1.11 (0.82, 1.51) | 0.484 |
|  | Use of nephrotoxic antibiotics^f^ | | 1.13 (0.79, 1.61) | 0.506 |
|  | Use of NSAIDs | | 1.19 (0.88, 1.60) | 0.257 |
| Postoperative laboratory (POD 0-3) and clinical outcomes | | | |  |
|  | Maximum Cl^-^ (mmol l^-1^) | | 1.02 (0.99, 1.05) | 0.270 |
|  | Hyperchloremia in POD 0 (Cl^-^ ≥ 110 mmol l^-1^) | | 1.48 (1.01, 2.15) | 0.044 |
|  | Hyperchloremia in POD 0-3 (Cl^-^ ≥ 110 mmol l^-1^) | | 1.24 (0.89, 1.72) | 0.200 |
|  | Increases in Cl^-^ (mmol l^-1^) | |  |  |
|  |  | Q1 ≤ 1 (mmol l^-1^) | 1 | (0.968) |
|  |  | 1 < Q2 ≤ 3 (mmol l^-1^) | 0.97 (0.63, 1.49) | 0.889 |
|  |  | 3 < Q3 ≤ 6 (mmol l^-1^) | 0.99 (0.67, 1.46) | 0.959 |
|  |  | Q4 > 6 (mmol l^-1^) | 1.08 (0.73, 1.60) | 0.712 |
|  | Postoperative metabolic acidosis in POD 0-3 | | 0.87 (0.53, 1.42) | 0.567 |

a: eGFR (mL·min^-1^·1.73·m^-2^) = 186 × (Creatinine)^-1.154^ × (Age)^-0.203^ (× 0.742 if female)

b: Intraoperative hypotension was defined as mean blood pressure <60 mmHg for >1 minute

c: Intraoperative fluid balance (%) = {[Total input fluid (L) – Total output fluid (L)] × 100} / weight on admission (kg)

d: Inotropes/vasopressors include norepinephrine, epinephrine, vasopressin, dobutamine, and dopamine.

e: Diuretics include mannitol and furosemide.

f: Nephrotoxic antibiotics include aminoglycoside, cephalosporin, vancomycin, and sulfonamide.

AKI, acute kidney injury; ICU, intensive care unit; ASA classification, American Society of Anesthesiologists classification; eGFR, estimated glomerular filtration rate; RRT, renal replacement therapy; POD, postoperative day; NSAID, nonsteroidal anti-inflammatory drug
