# Supplementary material for: Hyperchloremia and postoperative acute kidney injury: a retrospective analysis of data from the surgical intensive care unit
Source: Crit Care. 2018 Oct 30;22:277. doi: 10.1186/s13054-018-2216-5 (PMC6206638; doi:10.1186/s13054-018-2216-5)
Supplement: Supplementary file 5 — Association of total fluid use with perioperative serum chloride levels in surgical ICU patients. Results of the Pearson correlation analysis between total fluid use and maximum serum chloride levels or increase in serum chloride levels during PODs 0–3. (DOCX 31 kb) [file 13054_2018_2216_MOESM5_ESM.docx]

File name: **Additional file 5**

File format: .docx

Title of data: Association of total fluid use with perioperative serum chloride levels in surgical ICU patients

Description of data: Results of the Pearson correlation analysis between total fluid use and maximum serum chloride levels or increase in serum chloride levels during PODs 0–3.

|  | | Maximum serum chloride  in POD 0-3 (mmol L^-1^) | Increase in serum chloride in POD 0-3 (mmol L^-1^) |
| --- | --- | --- | --- |
| Total fluid use in POD 0-3 (ml kg^-1^) | |  |  |
|  | NaCl 0.9% | 0.22* | 0.07* |
|  | NaCl 0.45% | 0.11* | 0.23* |
|  | Balanced crystalloid | -0.42* | 0.11* |
|  | Hydroxyethyl starch | 0.28* | 0.12* |
|  | Free water containing dextrose | -0.10* | 0.11* |

**P* < 0.01

Values represent Pearson correlation coefficients.

ICU, intensive care unit; POD, postoperative day
